# Prevention of nosocomial bacteremia associated with Staphylococcus aureus in Benin

**DOI:** 10.1186/2047-2994-4-S1-P255

**Published:** 2015-06-16

**Authors:** TA Ahoyo, AK Aissi, S Assavedo, TB Orou Yorou, VT Dougnon, DA Kinde Gazard

**Affiliations:** 1 Direction Nationale de la Santé Publique, Point Focal Sécurité Patient/DNSP/Ministère Santé Bénin, Benin; 2 Direction Nationale de la Santé Publique, Benin; 3 Direction Nationale des Etablissement Hospitalier, Ministère de la Santé, Benin; 4 GBH, EPAC/University, Benin; 5 cabinet, Ministère de la Santé, Cotonou, Benin

## Introduction

Nosocomial bacteremia due to *Staphylococcus aureus* seem to be increasing in some hospitals in Benin according the first nationwide survey on the prevalence of nosocomial infection

## Objectives

Reduce the rate of nosocomial bacteremia in order to assess the importance of an effective infection program that could lead to prevent the spread of this bacterium

## Methods

From 1^st^ February 2013 to 30^th^ May 2014, according to WHO’s guidelines, hospital surveillance about nosocomial bacteremia has been conducted. Management of the study included a rigorous attention to the placement and the maintenance of vascular devices and correct hand hygiene. Two categories of tertiary hospitals, one with effective infection control program (category I) and the other without structured program (category II) were included

## Results

A total of 12 hospitals were involved in this study. Five of them had an effective infection control program. Nosocomial bactereamia incidence rate was 1.42 cases per 1000 patient per days in category I versus 4.83 cases per 1000 patient per days for category II. The proportion of S. *aureus* bacteremia was 5.2 % in category I versus 17.2 % in category II. The incidence of bacteremia began to decrease on January 2014, the primary site was discernible in 90% of bacteremia episodes only in hospitals category I.

## Conclusion

This study showed a significative reduction in the incidence of *S. aureus* bacteremia in hospital that had an effective infection program based on African Partnership Patient Safety Program

## Disclosure of interest

None declared.
